# Quantitative Structure-Activity Relationship Model for HCVNS5B inhibitors based on an Antlion Optimizer-Adaptive Neuro-Fuzzy Inference System

**DOI:** 10.1038/s41598-017-19122-y

**Published:** 2018-01-24

**Authors:** Mohamed Abd Elaziz, Yasmine S. Moemen, Aboul Ella Hassanien, Shengwu Xiong

**Affiliations:** 1 School of Computer Science and Technology, Wuhan University of Technology, Wuhan, China; 2 Department of Mathematics, Faculty of Science, Zagazig University, Zagazig, Egypt; 3 Clinical Pathology Department, National Liver Institute, Menoufia University, Menofia, Egypt; 4 Faculty of Computers and Information, Cairo University, Giza, Egypt

## Abstract

The global prevalence of hepatitis C Virus (HCV) is approximately 3% and one-fifth of all HCV carriers live in the Middle East, where Egypt has the highest global incidence of HCV infection. Quantitative structure-activity relationship (QSAR) models were used in many applications for predicting the potential effects of chemicals on human health and environment. The adaptive neuro-fuzzy inference system (ANFIS) is one of the most popular regression methods for building a nonlinear QSAR model. However, the quality of ANFIS is influenced by the size of the descriptors, so descriptor selection methods have been proposed, although these methods are affected by slow convergence and high time complexity. To avoid these limitations, the antlion optimizer was used to select relevant descriptors, before constructing a nonlinear QSAR model based on the PIC_50_ and these descriptors using ANFIS. In our experiments, 1029 compounds were used, which comprised 579 HCVNS5B inhibitors (PIC_50_ < ~14) and 450 non-HCVNS5B inhibitors (PIC_50_ > ~14). The experimental results showed that the proposed QSAR model obtained acceptable accuracy according to different measures, where ${{\boldsymbol{R}}}^{{\boldsymbol{2}}}$ was 0.952 and 0.923 for the training and testing sets, respectively, using cross-validation, while ${{\boldsymbol{R}}}_{{\boldsymbol{L}}{\boldsymbol{O}}{\boldsymbol{O}}}^{{\boldsymbol{2}}}$ was 0.8822 using leave-one-out (LOO).

## Introduction

Hepatitis C virus (HCV) is a member of the Flaviviridae family and it comprises six major genotypes, with a huge number of subtypes in each genotype^1^. The HCV genotype distributed throughout the world include genotype 1 (Japan, Europe, and North America), genotype 2 (Japan and North America), genotype 3 (Indian subcontinent), genotype 4 (North Africa and the Middle East), genotype 5 (South Africa), and genotype 6 (South East Asia)^2,3^.

The global prevalence of HCV is about 3%^4,5^ and one-fifth of all HCV carriers live in the Middle East^2^. About 20% of Egyptians are estimated to have HCV infections with almost half a million infections per year^6,7^. Thus, Egypt has the highest global incidence of HCV infection^8,9^, which varies from 9% to 50% in some rural areas due to the specific modes of infection^5^. The mechanism of infection has been fully elucidated, but viral entry and replication are not completely understood^7^. HCV possesses different enzymes and it has been suggested that HCV polymerase is the main enzyme involved in the viral replication process^10^.

Similar to other DNA polymerases, the architecture of HCV NS5b polymerase resembles a right hand with “thumb”, “palm” and “fingers” domains. The palm domain catalyzes the phosphoryl transfer reaction, whereas the fingers domain participates in interactions with the incoming nucleoside triphosphate as well as the template base with which it is paired^10^. HCV NS5b is an interesting target for antiviral therapy with limited side effects and it has been the subject of extensive trials to design nucleoside and non-nucleoside inhibitors^11^.

In recent years, the quantitative structure-activity relationship)QSAR(model has attracted much attention in pharmaceutical research because it can produce high-quality leads in the early stages of drug discovery^12^. In addition, QSAR reduces the costs of experiments and the failure rate when identifying lead compounds. Hansch *et al*. were the first to apply QSAR more than 50 years ago^13^, but Cros actually developed the QSAR concept in the toxicology field in 1863 by establishing a relationship between the toxicity of alcohols and their water solubility^14^. Thus, QSAR was developed in physical chemistry, before being applied to data sets containing many thousands of compounds with varied molecular structures, while it also evolved from modest regression approaches to wide-ranging statistical and machine learning methods. The QSAR technique has been used extensively in academic research, industry, and governmental institutions throughout the world. QSAR methods are employed to guide lead optimization approaches^15^. QSAR models can be applied broadly to evaluate the potential effects of chemicals, materials, and nanomaterials on human health and the environment.

In general, QSAR is used to determine the relationship between the chemical structural features (molecular descriptors) of compounds and their biological activity by using mathematical equations. For example, Hansch *et al*. proposed a relationship between lipophilicity and biological potency based on a study by Veldstra^16^. Fujita *et al*. used quantum-chemical calculations to measure the differences in activity regulation in growing plants^17^, where the experimental value of the octanol–water property (logP) was measured. Other studies have employed computational approaches to determine different effects of substituents on potency^18,19^.

Dimensionality can be used to define the type of QSAR used according to the chemical structure dimension. one-dimensional (1D)-QSAR describes the activity based on the total molecular properties such as logp and constitutional properties (no. of atoms of oxygen, nitrogen, etc.). 2D-QSAR associates the activity with the chemical structure such as a pharmacophore.3D-QSAR relates the activity to the interaction fields of molecules. 4D-QSAR is represented by using a group of 3D-ligand conformations, 5D-QSAR considers diverse induced-fit models of 4D-complexes, and 6D-QSAR includes various solvation models^20^.

QSAR models can be calculated using the following two groups of methods^21^. The first group comprises linear methods such as linear regression, partial least-squares, multiple linear regression, and principal component analysis (PCA). However, these methods are affected by various limitations, e.g., the interactions between the dependent and independent variables cannot be interpreted easily due to nonlinear relationships. In contrast to linear methods, the second group comprising nonlinear QSAR methods can determine the nonlinear mappings based on physicochemical and biological descriptors of the molecules^22^^,^^23^ while they also avoid some drawbacks of the linear QSAR methods. The nonlinear QSAR methods include artificial neural networks (ANNs), k-nearest neighbors, Bayesian neural nets^20^, fuzzy mappings^24^ and the adaptive neuro-fuzzy inference system (ANFIS)^25^.

ANNs have been applied to determine activity levels^26^, but this method is sensitive to the parameters and it can be trapped by local optima. Therefore, other methods may be used such as support vector machines (SVMs)^27^ and ANFIS^28^. ANFIS is a combination of ANN and fuzzy logic systems, which exploits the advantages of both (i.e., good reasoning using fuzzy logic systems and the simplicity of ANN), and it obtains good results for regression problems (especially the QSAR problem)^29^.

However, ANFIS has some limitations because it is influenced by the approach used to learn its parameters. In addition, a large number of descriptors may affect the performance of ANFIS (or any other QSAR model). Therefore, selecting the best descriptors is very important for reducing the computational cost and eliminating irrelevant descriptors that might reduce the accuracy of ANFIS. Several methods can be used to select the optimal subset of descriptors, including a method^25^ comprising two stages where the first employed a genetic algorithm (GA) to select suitable descriptors for the inhibitory activity of cathepsin K, before ANFIS was then used to predict the bioactivity values for cathepsin K. PCA has also been used to determine the largest eigenvectors representing the best descriptors, where these descriptors were then used as inputs for ANFIS and SVM to predict biologically active catechol structures^21^. However, most descriptor selection approaches, such as GA, are affected by limitations such as slow convergence and time complexity, while PCA and other dimension reduction methods change the original data set. Therefore, the antlion optimizer (ALO) was proposed to solve this problem^30^. ALO is a recently developed swarm intelligence technique, which emulates the natural interactions between antlions and ants. The ALO algorithm has several advantages such as small number of parameters, free gradient and good ability to balance between the exploration and exploitation^30^. Therefore, it has been applied in several applications such as, Esha Gupta *et al*. have proposed method based on ALO algorithm to determine the optimal parameters of primary governor loop of thermal generators, also it used to solve the process planning and scheduling functions in the manufacturing process^31^, and to find the optimal sizing and location of renewable distributed generations^32^. In addition, it has been used in several power system problem for example, load forecasting^33^, economic power dispatch^34^, and load frequency control^35^.

According to the previous literature, in this study, we developed an accurate, simple, reliable, and less computationally expensive technique for calculating bioactivity values by combining the ALO algorithm with ANFIS. The proposed model, called ALO-ANFIS, comprises two phases. In the first phase, the ALO algorithm was used to determine relevant descriptors. In the second phase, the ANFIS method was used in QSAR for modeling the relationships between the bioactivities of 1029 HCVNS5B compounds and their structural descriptors (those selected by ALO). In order to evaluate the performance of the selected descriptors, the data set was split into training and testing sets, where the training set comprised 772 compounds for refining the model and the testing set comprised 257 appropriately selected chemicals for testing the model. The accuracy of the ALO-ANFIS model was assessed using leave-one-out (LOO), Y-randomization, and external validation techniques.

The remainder of this paper is organized as follows. In Section 2, we introduce the methods and provide a brief discussion of the data set and their chemical descriptors, as well as the approach used for splitting the data and the basic concepts employed in ANFIS and ALO. In Section 3, we explain the proposed QSAR model. The experimental results are presented in Section 4. In Section 5, we give our conclusions and suggestions for future research.

## Methods

### Data sets and chemical descriptors

Chemical descriptors define the construction and function of designated chemicals. Many descriptors are now easy to calculate due to advances in computer technology^36^. These descriptors are classified according to several types such as compositional descriptors, topological descriptors, quantum descriptors, electronic parameters, and geometric parameters. Descriptors may be integers, such as the molecular weight, which describes the entire compound, or substitutions such as a steric effect constant, which refers to a precise fragment or group. Integral descriptors are used only for specific compounds, whereas substitutional descriptors can be used for several compounds, e.g., trichloromethane and trichloroacetic acid have equal steric effect constant values for chlorine^37^.

**Figure 1:**
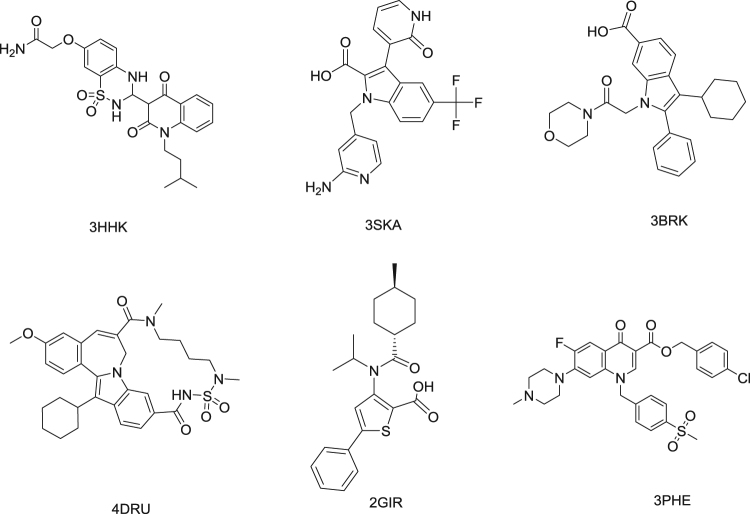
HCV inhibitors.

In the current work, Six HCV inhibitors (Fig. 1) (PDB ID: 3HHK, 3SKA, 2BRK, 4DRU, 2GIR and 3PHE) with their derivatives which were collected from literature^38–43^ and gathered in the current dataset^44^. Their structures in smile format, PIC_50_ and their literature are represented in the supplementary information.

In this study, 1029 compounds were used to build the QSAR model, which comprised 579 HCV NS5B inhibitors, (PIC_50_ < ~14) and 450 non-HCVNS5B inhibitors (PIC_50_ > ~14)^45^. These compounds were collected from previous studies and extracted from the BindingDB and CHEMBL databases^10,46^. The chemical descriptors were calculated using the DataWarrior package^47^. Thus, DataWarrior was used to calculate the properties of compound such as the drug-likeness, atom counts, and functional groups, where these descriptors represented 29 properties, as shown in Table 1.

**Table 1:** Description of the data set used in this study.

No. Feature	Features	No. Feature	Features
1	Absolute Weight	16	Rings
2	cLogP(Octanol/Water, partition coefficient)	17	Aromatic Rings
3	cLogS (Aqueous solubility)	18	Aromatic Atoms
4	H-Acceptors (Hydrogen bond Acceptor)	19	sp3-Atoms
5	H-Donors (Hydrogen bond Donor)	20	Symmetric atoms
6	Total Surface Area	21	Amides
7	Polar Surface Area	22	Amines
8	Druglikeness	23	Alkyl-Amines
9	Shape Index	24	Aromatic Amines
10	Molecular Flexibility	25	Aromatic Nitrogens
11	Molecular Complexity	26	Basic Nitrogens
12	Non-H Atoms	27	Acidic Oxygens
13	Non-C/H Atoms	28	Stereo Centers
14	Metal-Atoms	29	Rotatable Bonds
15	Electronegative Atoms		

### Data splitting

In order to evaluate the performance of the proposed model, the training and testing sets were determined using the cross-validation method. In the cross-validation, the data set was split into a number of classes, before selecting one class as the testing set, whereas the other classes were used to construct the training set. We performed a 10-fold cross validation, with nine classes representing the training set and one representing the testing set, where this process was performed 10 times and the average accuracy was calculated based on all the runs.

### Adaptive Neuro-Fuzzy Inference System

The ANFIS method combines fuzzy logic and ANN^48,49^. In recent years, ANFIS has attracted much attention because of its many applications to renewable energy^50^ and wind prediction^51^.

In general, the Takagi–Sugeno inference method is one of most popular methods used in ANFIS^48^. This method generates a nonlinear mapping of fuzzy rules from the input space to the output space by using a number of fuzzy IF–THEN rules. The definition of the first-order Sugeno type is given by Eq. (1)^45^:

1 $$\begin{array}{c}({\rm{Rule}}\,{\rm{1}})\quad {\rm{IF}}\,x\,{\rm{is}}\,{Q}_{1}\quad {\rm{AND}}\quad y\,{\rm{is}}\,{P}_{1},\quad {\rm{THEN}}\,{f}_{1}={l}_{1}x+{m}_{1}y+{n}_{{\rm{1}}}\quad {\rm{and}}\\ ({\rm{Rule}}\,{\rm{2}})\quad {\rm{IF}}\,x\,{\rm{is}}\,{Q}_{2}\quad {\rm{AND}}\quad y\,{\rm{is}}\,{P}_{2},\quad {\rm{THEN}}\,{f}_{2}={l}_{2}x+{m}_{2}y+{n}_{2},\end{array}$$

where *Q*_1_, *Q*_2_ and *P*_1_, *P*_1_, *P*_2_ are the member functions (MFs) for *x* and y, respectively, and *l*_*i*_, *m*_*i*_, and *n*_*i*_ (*i* = 1, 2) are the associated parameters of the output functions.

**Figure 2:**
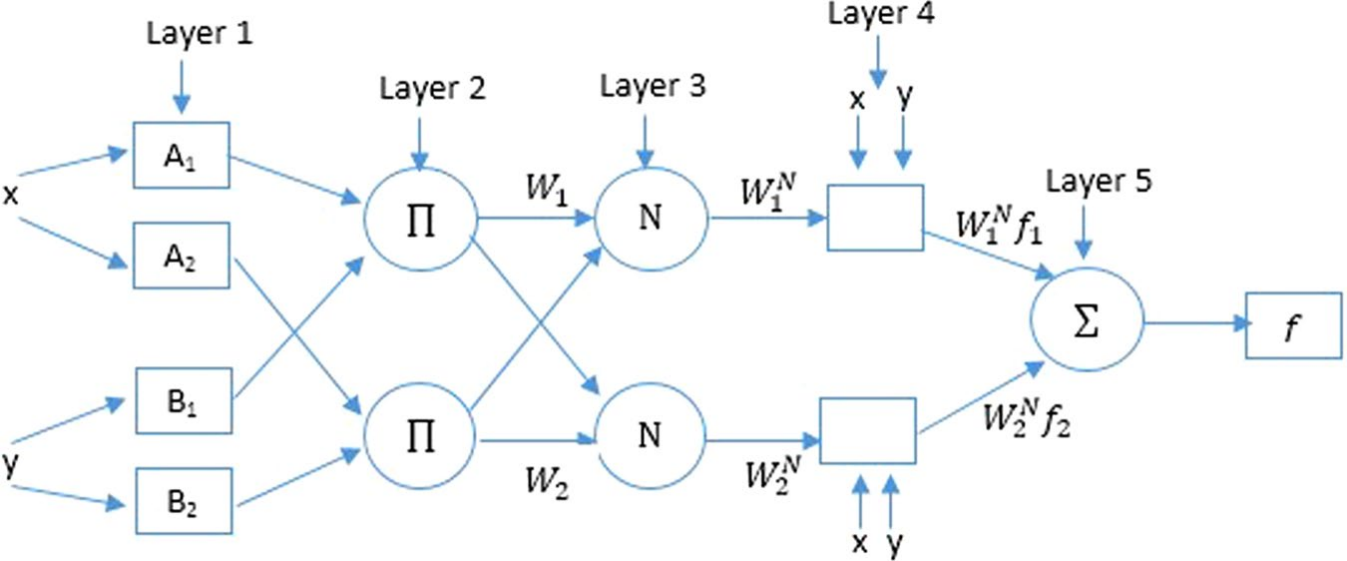
ANFIS layers.

The structure of the ANFIS model comprises five layers (Fig. 2), where each layer represents part of the fuzzy inference system (FIS). In particular, the first layer represents the fuzzification process, the second layer is responsible for computing the inference rules and their firing strengths, the third layer is the normalization layer, the adoption layer in FIS is represented by the fourth layer, and the defuzzification output is represented by the last layer. The details of each layer are given in the following. In the structure of ANFIS, the FIS parameters are encoded as weights between layers in the ANN, which allows optimization methods (used in ANN) to be used to determine the optimal FIS parameters.

The first layer receives the input data (*x* and *z*) at node *i* and then computes the membership values (*A*_*i*_ and *B*_*i*_) of the MFs as the output of each node, as given by Eq. (2):

2 $${O}_{1i}={\mu }_{{A}_{i}}(x),i=1,2,{O}_{1i}={\mu }_{{B}_{i-2}}(y),i=3,4,$$

where *μ*_*A*_ and *μ*_*B*_ are the MFs defined as:

3 $$\mu (x)={e}^{-{((x-{\rho }_{i})/{\sigma }_{i})}^{2}},$$

where *ρ*_*i*_ and *σ*_*i*_ are the data mean and standard deviation, respectively, which represents the premise parameters set (note that Eq. (3) is the generalized Gaussian MF)^48^.

The second layer computes the firing strength of a rule (*ω*_*i*_) by each node as follows.

4 $${\omega }_{i}={\mu }_{{A}_{i}}(x)\times {\mu }_{{B}_{i-2}}(y)$$

The third layer computes the normalization of the firing strength $({\bar{w}}_{i})$ as:

5 $${\bar{w}}_{i}=\frac{{\omega }_{i}}{{\sum }_{i=1}^{2}{\omega }_{i}}.$$

The fourth layer nodes compute the output using the following equation:

6 $${O}_{4,i}={\bar{w}}_{i}\,{f}_{i}={\bar{w}}_{i}({l}_{i}x+{m}_{i}y+{n}_{i}),$$

where *l*_*i*_, *m*_*i*_, and *n*_*i*_ are the consequent parameters for node *i*.

The final layer computes the output of the whole model where this layer comprises a single node:

7 $${O}_{5}={\sum }_{i}{\bar{w}}_{i}\,{f}_{i}.$$

The ANFIS model employs two sets of adjustable parameters: the premise and consequent parameters, which need to be updated throughout the learning phase until the optimal global values are achieved based on the desired response.

### Antlion Optimizer

The Antlion Optimizer (ALO) algorithm starts by generating two random populations of antlions and ants, before assessing each position in these two populations using a fitness function, and the optimal ant position is then found^30^. In general, the ants update their solution based on the antlions, which are selected by random walk or according to an elite individual. Each ant’s position is updated based on the antlion selected by random walk, where the random walks are normalized according to the following equation^30^:

8 $${X}_{i}^{t}=\frac{({X}_{i}^{t}-{a}_{i})\times ({d}_{i}-{c}_{i}^{t})}{({d}_{i}^{t}-{a}_{i})}+{c}_{i},$$

where ${a}_{i},{b}_{i},{c}_{i}^{t},$ and ${d}_{i}^{t}$ represent the minimum and maximum of the random walk, and the minimum and maximum of the *i*-th variable in the *t*-th iteration, respectively.

The antlion traps affect the random walks of ants, so the ants move within a hyper-sphere (defined by *c* and *d*) around a selected antlion $Antlio{n}_{j}^{t}$. This process is emulated by the ALO algorithm as follows^30^:

9 $${c}_{i}^{t}=Antlio{n}_{j}^{t}+{c}^{t}$$

10 $${d}_{i}^{t}=Antlio{n}_{j}^{t}+{d}^{t},$$

where *d*^*t*^ and *c*^*t*^ are the maximum and minimum of all the variables, respectively.

The next process is defined as trap building where the roulette wheel method is used to select $Antlio{n}_{j}^{t}$ based on a fitness function. Thus, a fitter antlion has a higher likelihood of catching the ants.

After building traps, the antlions shoot sands outward from the pit center when ants are in the trap. This behavior is emulated by making *c*^*t*^ and *d*^*t*^ (defining the radius of the hyper-sphere of ant random walks) decrease with respect to the current iteration *t* as:

11 $${c}^{t}=\frac{{c}^{t}}{I},{d}^{t}=\frac{{d}^{t}}{I},I=\frac{{10}^{w}t}{T},$$

where *w* is a constant for adjusting the exploitation performance and its value is given as: (1) *w* = 2 when *t* > 0.1 *T*, (2) *w* = 3 when *t* > 0.5 *T*, (3) *w* = 4 when *t* > 0.75 *T*, (4) *w* = 5 when *t* > 0.9 *T*, and (5) *w* = 6 when *t* > 0.95 *T*.

When the ant prey reaches the bottom of the pit, the antlion catches it and takes it into the sand to begin eating. The mathematical definition of this process is given as:

12 $$Antlio{n}_{j}^{t}=An{t}_{i}^{t}\,if\,f(An{t}_{i}^{t}) > f(Antlio{n}_{i}^{t}),$$

where $An{t}_{i}^{t}$ is the position of the *i*-th ant in the *t*-th iteration. This equation considers that antlions catch prey if they are fitter than other antlions. The position of the antlion is updated to the latest position of the hunted ant to enhance its chance of catching new prey.

In addition to selecting an antlion by random walk, it can be determined using an elite individual strategy, where the best position is used to represent the elite individual. The movement of each ant around a selected antlion may be selected simultaneously by the roulette wheel method and the elite individual strategy, which is formulated as^30^:

13 $$An{t}_{i}^{t}=\frac{{R}_{A}^{t}+{R}_{E}^{t}}{2},$$

where ${R}_{A}^{t}$ and ${R}_{E}^{t}$ are the random walk around the antlion selected by the roulette wheel and the best position selected by the elite individual strategy, respectively.

## Proposed QSAR model

In this section, we explain the proposed QSAR model for predicting the activity of HCV NS5B inhibitors and non-inhibitors. This approach is called the ALO-ANFIS QSAR model and it comprises two phases, where the first is the descriptor selection phase and the second is the PIC_50_ prediction phase, as given by Algorithm 1. The details of each phase are explained in the following subsections, where the first step in the proposed algorithm is dividing the HCV NS5B data set into training and testing sets using suitable method (such as 10-fold cross-validation method).

### Feature selection phase

In this phase, the ALO algorithm is used to select the most relevant features from the training set as follows.

The ALO algorithm starts by generating two populations of ants and antlions, which each solution is converted into binary vector (representing the selected features) using the following equation^52^:

14 $${x}_{ij}=\{\begin{array}{c}1\,{x}_{ij}\ge \varepsilon \\ \,\,0\,otherwise\end{array},$$

where *ε* ∈ [0, 1] is a threshold and *x*_*ij*_ represents the *j*th feature of *x*_*i*_. For example, if *x*_*i*_ = 01100, then the second and third features are selected.

The fitness function *f*_*i*_ is calculated for each solution as:

15 $${f}_{i}=\alpha \times \sqrt{\frac{1}{{N}_{\mbox{--}}S}\sum _{i=1}^{{N}_{\mbox{--}}S}{({\hat{Y}}_{i}-{Y}_{i})}^{2}}+(1-\alpha )\times (\frac{|x|}{D}),$$

where α ∈ [0, 1] is a random number, and |*x*| and *D* are the number of selected descriptors and the total number of features, respectively. The ${\hat{Y}}_{i}$ and *Y*_*i*_ represent the predict and the actual value of PIC_50_, respectively, for the training set. Also, *N*_*S* is the total number of samples in the PIC_50_. The fitness function considers the root mean squared error (RMSE) and the number of selected descriptors with the aim of minimizing both.

Based on the best fitness function, the elite individual is selected as the best position for the antlion, and each ant (from the population of ants) updates its position based on the antlion selected using the roulette wheel method. Next, *c* and *d* defining the radius of the hyper-sphere are updated, before updating the position of each ant based on the random walk around the selected antlion and the elite individual. The positions of the ants are evaluated and their fitness values are compared with those of the antlions, before the antlions replace their position with those of the corresponding fittest ant. These steps are repeated until the maximum number of iterations is reached or when the difference between the two fitness function becomes smaller than a threshold.

### Regression phase

In the regression phase, the reduced training set (after select the features from the first phase) is used as the input for the ANFIS model where it is used to learn the ANFIS parameters. After the ANFIS training stage has finished, the testing set (with the same selected features) is used as the input and the ANFIS output is computed. The performance of the output is compared with the actual PIC_50_ value, where the RMSE and other measures are computed.

**Algorithm 1:** ALO-ANFIS QSAR model.

1. Input: Data set for QSAR *D*, and the target *y*_*IC*50_
2. Output: Predicted values and performance measures.
3. Define: Number of solutions *N* in the population of ants and antlions, *Iter*_*max*_ as the maximum number of iterations, and *c*, *d* defining the radius of the hyper-sphere
4. *Divided the dataset D into training (D*_*train*_*) and testing* (*D*_*test*_) *sets using 10-fold CV method*.
*Feature Selection Phase:*
5. *X* = ALO(*Iter*_*max*_, *N*, *c*, *d*, *D*_*train*_)
*Regression Phase*
6. Create new training set based on the selected features *D*_*newtrain*_ = *D*_*train*_(:,*X*)
7. Learn the ANFIS parameters based on the training set to obtain the (*trained model* = ANFIS(*D*_*newtrain*_)).
8. Create new testing set based on the selected features *D*_*newtest*_ = *D*_*test*_(:, *X*)
9. Apply the trained model to the testing set to compute the predicted value of PIC_50_ (${\hat{y}}_{IC50}={\rm{Trained}}\,{\rm{model}}({D}_{newtest})$)
10. Evaluate the performance of the predicted output ${\hat{y}}_{IC50}.$

**Algorithm 2:** ALO algorithm (*Iter*_***max***_, *N*,*c*, *d*, *D*).

1. Generate a random two populations of ants and antlions.
2. For *i* = 1: *N*
3. Evaluate the fitness *fAL*_*i*_ of *antlions*_*i*_ using Eq. (15).
4. End
5. *t* = 1 //initial value of the current iteration
6. Do
7.  Determine the best solution (Elite) by selecting the best *fAL*_*best*_.
8.  For *i* = 1: *N*
9. Choose the antlion using the roulette wheel method (*antlions*_*RW*_).
10. Update the radius of the hyper-sphere, *c* and *d*, using Eqs. (9)–(11).
11. Use Eq. (8) to perform a random walk around *antlions*_*RW*_ with the roulette wheel method $\,{R}_{A}^{t}$.
12. Use Eq. (8) to perform a random walk around the elite individual ${R}_{E}^{t}$.
13. Use Eq. (13) to update the position of *ant*_*i*_.
14. Evaluate the fitness *fant*_*i*_ of *ant*_*i*_ using Eq. (15).
15. If *fant*_*i*_ ≤ *fAL*_*i*_
16. * antlions*_*i*_ = *ant*_*i*_ (using Eq. (12))
17. End IF
18. End For
19. *t* = *t* + 1
20.  Until (*t* < *Iter*_*max*_)
21. Return elite *X*

**Algorithm 3:** ANFIS(*D*_*tain*_).

1. Normalize the training set (*D*_*tain*_) to be *D*_*norm*_.
2. Construct the ANFIS network with Gaussian MF.
3. DO
4. Compute the membership values for each node in the first layer
5. Computes the firing strength of the rule for each node in the second layer
6. Compute the normalized firing strength in the third layer.
7. Compute the output for each node in the fourth layer using Eq. (6).
8. Compute the output $\hat{y}$ using Eq. (7).
9. If the difference between the $\hat{y}$ and *y* is smaller than *ε*,
10.  Break
11. Else
12.  Update the parameters of the ANFIS model using the backpropagation method.
13. End
14. *t* = *t* + 1
15. Until the maximum number of iterations is reached.
16. Return the trained model

### The complexity of the proposed QSAR model

The computational complexity of the proposed ALO-ANFIS is depended on some elements (1) the population size (N). (2) Maximum number of iterations (*Iter*_*max*_), (3) the number of features (N_*f*_), (4) sample size of the dataset (N_*s*_). (5) The number of cluster in ANFIS model (N_*C*_), number of selected features (N_*sf*_), (6) the sorting algorithm (we used the Quicksort).

Where the complexity of Quicksort in best case is *O*(*NlogN*) and in the worst case is *O*(*N*^2^). Therefore, the proposed ALO-ANFIS model has complexity

16 $$\begin{array}{l}O(ALO-ANFIS)=\{\begin{array}{l}(4N\times {{\rm{N}}}_{f}+O(N\,log\,N))\times Ite{r}_{max}+O({{\rm{N}}}_{s}\times {{\rm{N}}}_{sf}\times {{\rm{N}}}_{C})\times Ite{r}_{\mathop{\max }\limits_{ANFIS}}\,best\,case\\ (4N\times {{\rm{N}}}_{f}+O({N}^{2}))\times Ite{r}_{max}+O({{\rm{N}}}_{s}\times {{\rm{N}}}_{sf}\times {{\rm{N}}}_{C})\times Ite{r}_{\mathop{\max }\limits_{ANFIS}}\,worst\,case\end{array}\end{array}$$

## Experiments and Results

### QSAR Model

The experiments were implemented in Matlab and run in a 64-bit Windows environment. The parameters comprised a population size N = 25, maximum number of iterations = 100, and the stopping condition was reaching the maximum number of iterations. We evaluated the performance of the proposed model by changing the maximum number of iterations, but we found that when the maximum number of iterations exceeded 100, the increase in performance became very small as the computational time increased. In addition, when the maximum number of iterations was less than 100, the computational time was reduced but the performance of the proposed model was greatly degraded. The same effects were obtained when the size of the population was varied.

### Molecular docking

After filtration the 1029 chemical structures to reach 140 structures, molecular docking process was carried out to validate the QSAR technique, supplementary information.

The protein crystal structure, **3HHK**, were cleaned and treated as described in literature^53^; the crystal structure was used because of its high resolution 1.7 Å. Chimera was used for crystal structure preparation^54^, only one chain of **3HHK** protein was used, all ligands or any solvent molecules were discarded, and polar hydrogens were added by MGLTools.

The MTiOpenScreen is a virtual screening server, which uses Autodock Vina as a docking tool^55^ to investigate the highly potent and selective molecular compounds as mentioned before^56^. When using a big data set reaches to 1000 structures, a certain criteria will be applied to ensure a good absorption/penetration of the drugs. this criteria includes Lipenski rule of five (no more than 5 hydrogen bonds, no more than 10 hydrogen bond acceptors, the molecular weight less than or equal to 500 daltons and an octanol-water partition coefficient not be greater than 5) and other two criteria the rotatable bonds less than 10 and the polar surface area will be less than 140 Å^57^.

Molegro molecular view^58^ was used to extract the docked inhibitors from their receptors for the graphic representation.

### Performance measures

Three groups of measures were used to evaluate the performance of the proposed model. The first group comprised statistical measures for evaluating the performance by comparing the predicted output obtained from the proposed model and the actual value. The second group used a set of criteria to determine whether the proposed model obtained suitable predictions. The third group determined the applicable domain for the proposed model. Definitions of each of these measures are given in the following.

#### Statistical measures

- The mean squared error (*MSE*) measures the difference between the predicted value and the actual values as:
  17 $$MSE=\frac{1}{{N}_{\mbox{--}}S}\sum _{i=1}^{{N}_{\mbox{--}}S}{({\hat{Y}}_{i}-{Y}_{i})}^{2}.$$
- *RMSE* represents the data dispersion around zero deviation, which is defined by:
  18 $$RMSE=\sqrt{\frac{1}{{N}_{\mbox{--}}S}\sum _{i=1}^{{N}_{\mbox{--}}S}{({\hat{Y}}_{i}-{Y}_{i})}^{2}}.$$
- Coefficient of determination (*R*^2^) measures the goodness of fit between the results obtained by a method and the actual data. If the value of *R*^2^ is closer to 1, the correlation between the experimental and predicted values is better. *R*^2^ is defined by:

19 $${R}^{2}=1-\sum _{i=1}^{{N}_{\mbox{--}}S}\frac{{({Y}_{i}-{\hat{Y}}_{i})}^{2}}{{({Y}_{i}-{\bar{Y}}_{i})}^{2}}.$$

#### Predictive criteria

In addition, we used the criteria proposed by Tropsha *et al*.^59^ for determining whether a model is predictive (when they are satisfied) or not (when they are not satisfied), as follows:

20 $$\begin{array}{l}1)\,{R}_{LOO}^{2} > 0.5,\,2)\,{R}^{2} > 0.6,\,3)\,\frac{{R}^{2}-{R}_{O}^{2}}{{R}^{2}} < 0.1,\,5)\,\frac{{R}^{2}-{R^{\prime} }_{O}^{2}}{{R}^{2}} < 0.1,\\ 6)\,0.85 < k < 1.15\,or\,0.85 < K^{\prime}  < 1.15,\end{array}$$

where *R*^2^represents the regression correlation coefficient of between *Y* and $\hat{Y}$ (the actual values of PIC_50_ and their predicted values, respectively) in both sets (training and testing), ${R}_{O}^{2}$ (${R}_{O}^{\text{'}2})\,\,$represent the regression correlation coefficients between $\hat{Y}$ and *Y* (*Y*versus $\hat{Y}$) through the origin, and *K* and *K*'represent the slopes of the regression lines through the origin (for more information about these measures, please refer to Tropsha *et al*.^59^.

Moreover, in another study^60^, *R*^2^ to was modified to determine the difference between ${R}_{O}^{2}$ and ${R}_{O}^{\text{'}2}$ as:

21 $${R}_{m}^{2}={R}^{2}(1-|\sqrt{{R}^{2}-{R}_{O}^{2}}|),$$

where the model is considered to have good external predictability if the value of ${R}_{m}^{2} > 0.5$.

#### Applicable domain

Tropsha *et al*.^59^ used the Williams plot to measure the applicable domain (AD) for the QSAR model, where this type of graph plots leverage values against the standardized residuals. The leverage value *h*_*i*_ for the elements of the independent variable is computed as:

22 $${h}_{i}={x}_{i}^{T}{({X}^{T}X)}^{-1}{x}_{i},$$

where *X*is the trained model constructed from the training set and *x*_*i*_ is the principal component vector of the element considered.

The warning leverage value (h^*^) is computed by 3*K* + 1/*N*_*S*, where *K* represents the total number of independent variables. The probability of accordance between the predicted and experimental values is high for the elements (molecules) in the training set when *h* < h^*^. In addition, *h* > h^*^ indicates that this element will enhance the QSAR model when the element is present in the training set. However, if this element is in the testing set, this indicates that the predicted value is unreliable. If the residual of this element is low, then this element cannot be considered as an outlier. Therefore, in order to determine the applicable domain of the QSAR model, the standardized residual and the leverage must be considered at the same time.

### Molecular docking evaluation

**Figure 3:**
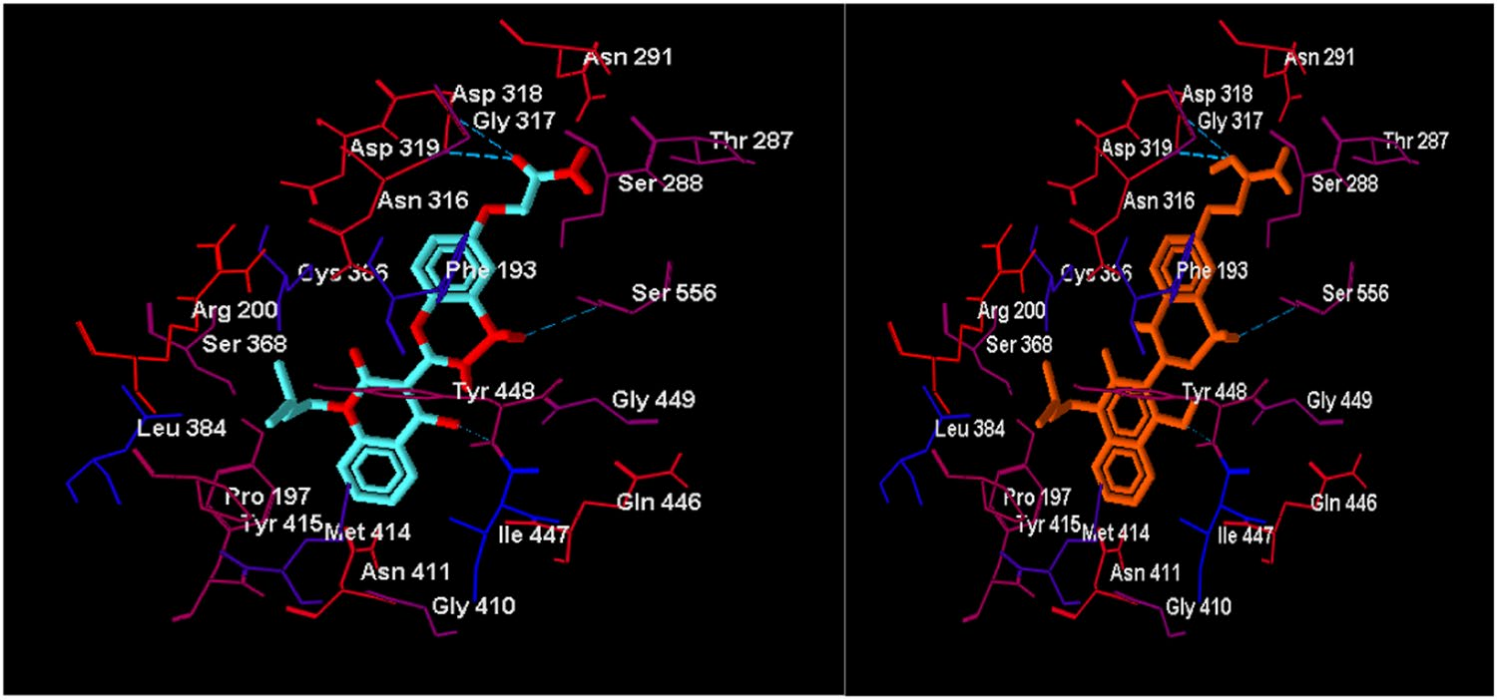
The structures of experimental (aqua) and docked structure (orange) to 3HHK receptor.

To validate the molecular docking of the current data set structures, the ligand **7ZZ** was re-docked to HCVNS5b or 3HHK as mentioned before, the RMSD between the experimental and docked **7ZZ** structure was less than 2 angstrom, see Fig. 3.

In Fig. 3, compound **7ZZ** colored in different colors to differentiate between experimental and docked structure. Amino acid residues appear as thin sticks while ligand atoms are represented as bold sticks. Hydrophilic residues have a red color, while hydrophobic residues have blue color. Atoms of residues are colored according to the hydropathy index proposed by Kyte and Doolittle in 1982^61^, the blue dashed line represents the hydrogen bond.

### Results and discussion

**Figure 4:**
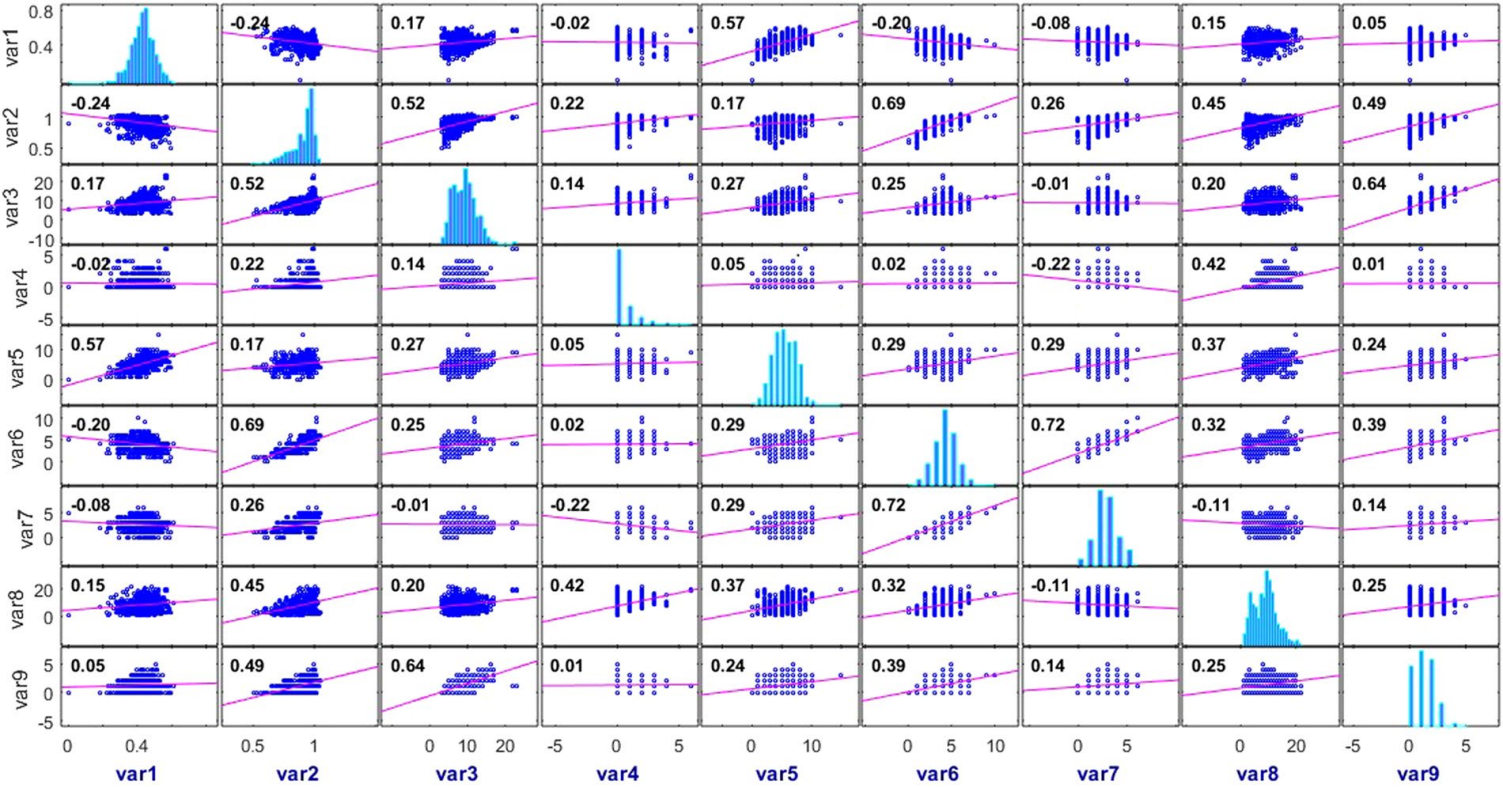
Correlation matrix.

The HCVNS5B QSAR model was built using the proposed ALO-ANFIS approach. The computed Pearson’s correlation coefficients between the selected descriptors (molecular flexibility (var1), molecular complexity (var2), non-C/H atoms (var3), electronegative atoms (var4), stereo centers (var5), rotatable bonds (var6), rings (var7), aromatic atoms (var8), and symmetric atoms (var9)) are shown in Fig. 4, which indicates there were positive and negative correlations between various descriptors, but the values were not high so no further descriptors were removed.

**Figure 5:**
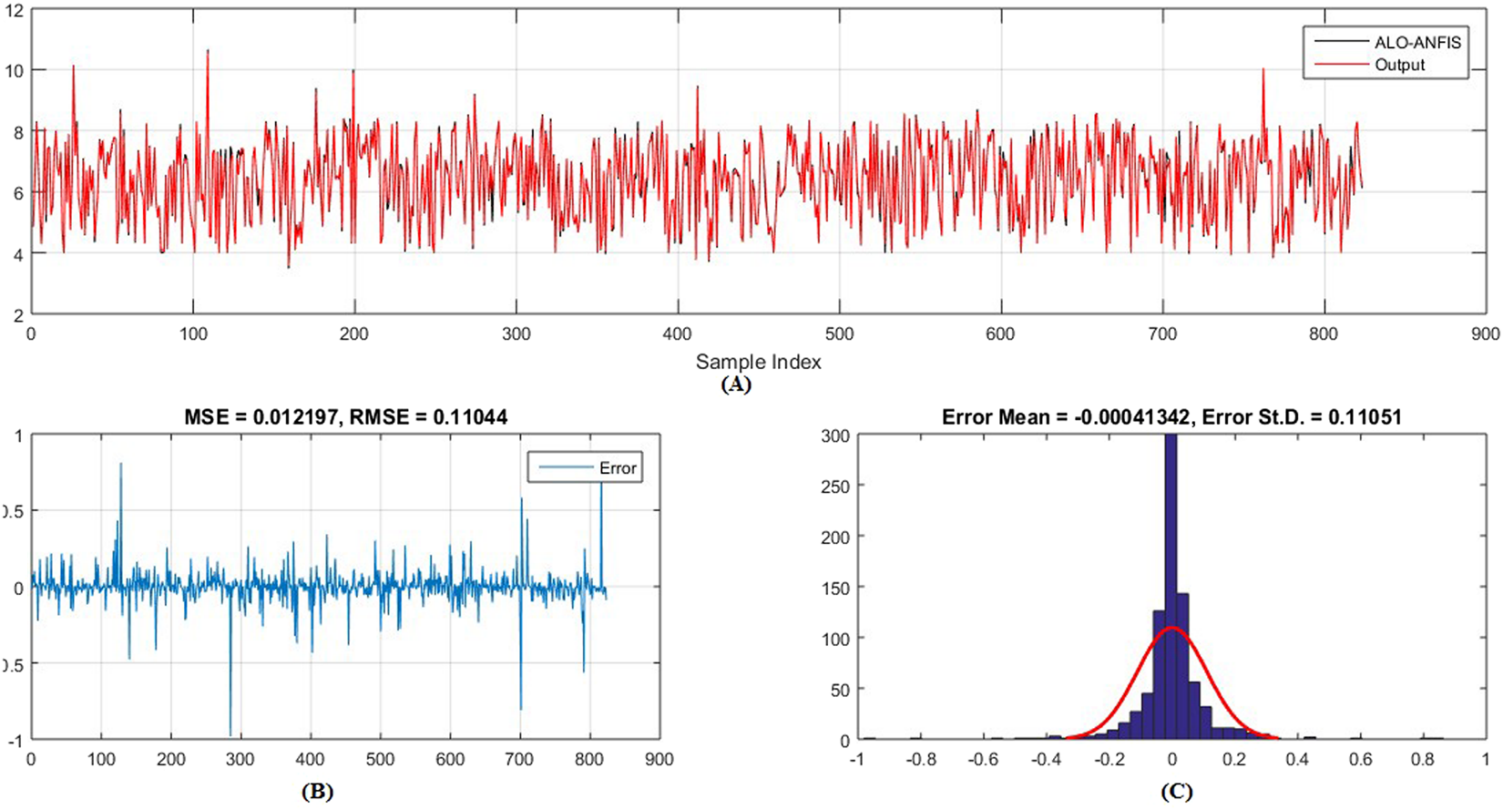
Training set results obtained by the proposed QSAR model (the output in the legend refers to the actual training set), (**A**) the predicted versus the actual, (**B**) the MSE and RMSE values, (**C**) the histogram of the Error.

**Figure 6:**
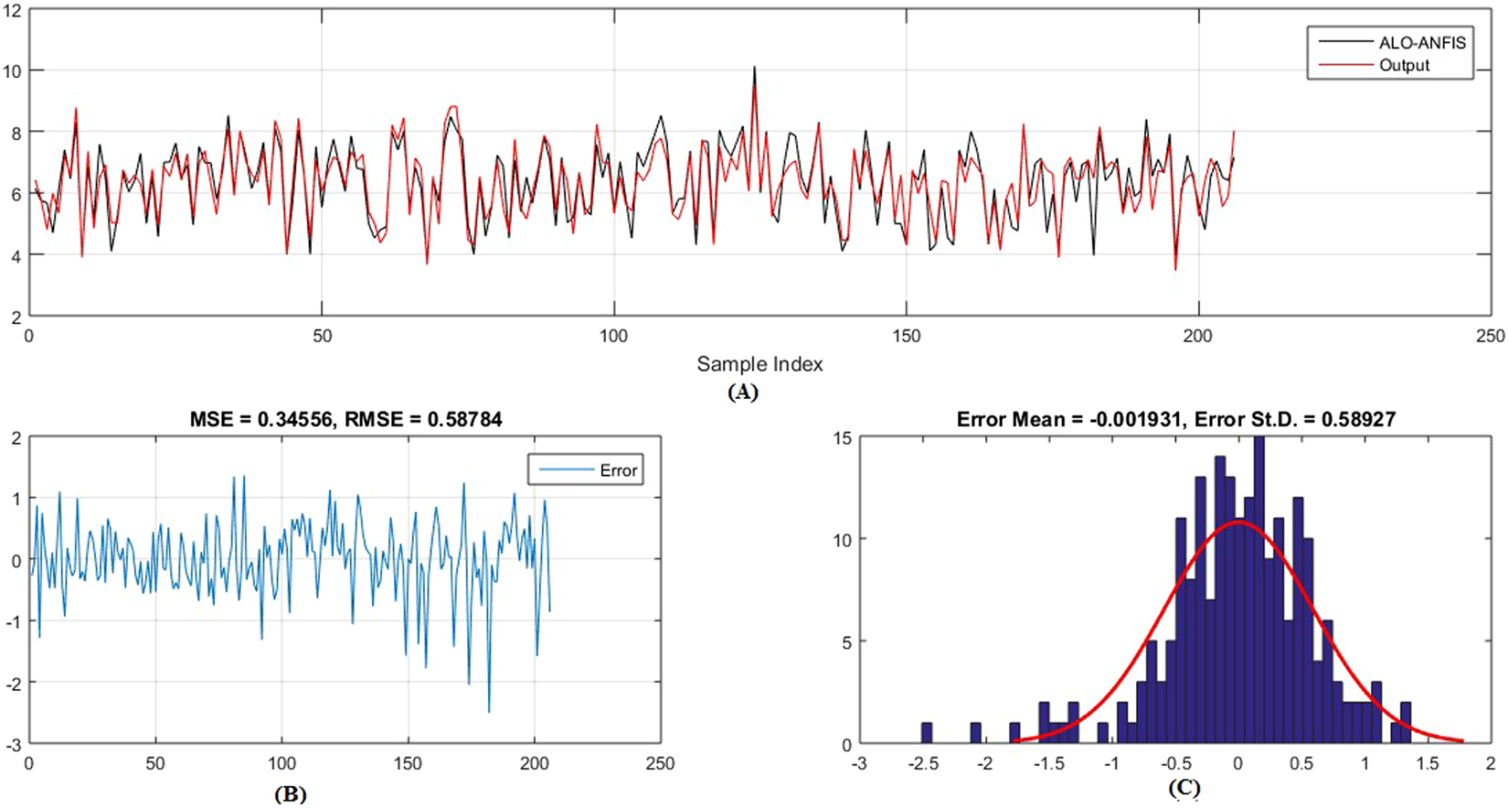
Testing set results obtained by the proposed QSAR model (the output in the legend refer to the actual testing set), (**A**) the predicted versus the actual, (**B**) the MSE and RMSE values, (**C**) the histogram of the Error.

**Figure 7:**
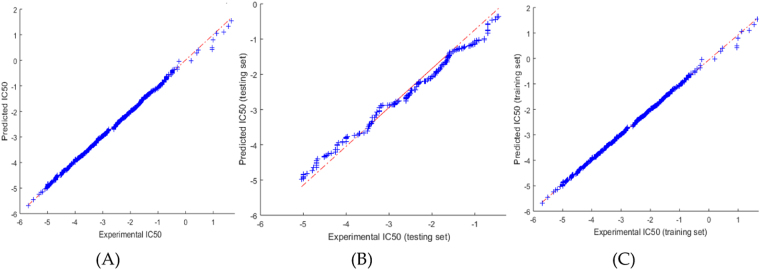
The Correlation results for the experimental PIC_50_ values versus the values predicted by the ALO-ANFIS model.

Figures 5–6 show the predicted values and the experimental values for the training set and testing set, respectively, which indicate that the predicted PIC_50_ values agreed well with the experimental values (prediction percentage error was less than 5%). The model did not exhibit proportional and systematic error because the distribution of the residuals to both sides of zero was random. In addition, Fig. 7 shows the regression plot for the training set, testing set, and all the actual PIC_50_ values.

Table 2 shows the results obtained for the proposed ALO-ANFIS model according to the set of measures, which indicates that the *RMSE*and *MSE* values were 0.1104 and 0.012, respectively. The value of ${R}_{var}^{2}\,$was higher than 0.6, but all of the results indicate that the goodness of fit for the proposed model was satisfactory. However, several QSAR models obtained good fits (such as ANN with a sufficient number of layers and neurons) but these models were not predictive.

**Table 2:** Statistical results obtained for the ALO-ANFIS model.

Statistics value	Training set	Testing set	Statistics value	Training set	Testing set
N	823	206	*MSE*	0.01219	0.346
*R* ^2^	0.952	0.923	*K*	0.972	0.849
*RMSE*	0.1104	0.588	*RMSELOO*	0.4440	
${R}_{LOO}^{2}$	0.8822		*R*_*m*_	0.759	0.566
*K*′	1.022	0.920	${R}_{var}^{2}$	0.993	0.774
${R}^{2}-{R}_{o}^{\text{'}2}/{R}^{2}$	−0.091	0.106	${R}^{2}-{R}_{o}^{2}/{R}^{2}$	−0.092	0.085

Therefore, several criteria must be satisfied in order to determine whether a model is predictive, as mentioned in the previous section. These criteria are divided into internal and external validation measures^59^. To assess the internal measures, the model was evaluated using only the available data (training) and no other external data (testing set) (e.g., the Y-randomization test). In contrast to the internal measures, the external measures depended on the testing data that had not been used already.

To test the predictability of the proposed model, we divided the data into a training set (823) and testing set (206) (which were selected randomly and they had not been used for training the model). The statistical measures obtained for the ALO-ANFIS model using the training and testing sets are given in Table 2. The RMSE of the proposed model was small for the testing set (~0.588), but after comparing this value with those reported previously^25,26^, we considered that this value was high, possibly because the testing set was larger than those used in previous studies. In addition, the *R*^2^ of values for the training and testing sets were 0.952, and 0.923, respectively, which are greater than 0.5.

Moreover, the results in Table 2 satisfy the criteria mentioned above^59^, where the ${R}^{2}$ value obtained for the testing set was greater than 0.6, and the ${R}^{2}$ value for the training set (or *Q*^2^) was greater than 0.5. In addition, the value of *R*^2^ was very close to ${R}_{0}^{2}$ (and ${R^{\prime} }_{0}^{2}),$ where the values of ${R}^{2}-{R}_{0}^{2}/{R}^{2}$ and ${R}^{2}-{R^{\prime} }_{0}^{2}/{R}^{2}$ were smaller than 0.11. Thus, based on the values of the ${R}_{m}^{2}$ parameter (the values were greater than 0.5 for both the training and testing sets), the ALO-ANFIS QSAR model was considered to be a predictive model.

We also obtained evidence that the ALO-ANFIS QSAR model is robust and predictive based on the chance correlation results produced using the LOO test and the Y-randomization test. The results of the LOO cross-validation for the training set (in Table 2) as ${R}_{LOO}^{2}\,\,$and *RMSE*_*LOO*_ were 0.8822 and 0.4440, respectively, so it is reasonable to use the ALO-ANFIS model in QSAR. In the Y-randomization test, we constructed a number of models (set to 10) by randomly permuting the data in the original model, and the expected values of *R*^2^ and *Q*^2^ for these constructed models were smaller than those for the original ALO-ANFIS QSAR model, as shown in Table 3.

**Table 3:** Results of the Y-randomization test.

Model	1	2	3	4	5	6	7	8	9	10
*R* ^2^	0.5006	0.4223	0.5828	0.4844	0.4931	0.4472	0.3987	0.4221	0.4758	0.4745
*Q* ^2^	0.8708	0.8691	0.8652	0.8627	0.8655	0.8818	0.8810	0.8675	0.4527	0.4615

**Figure 8:**
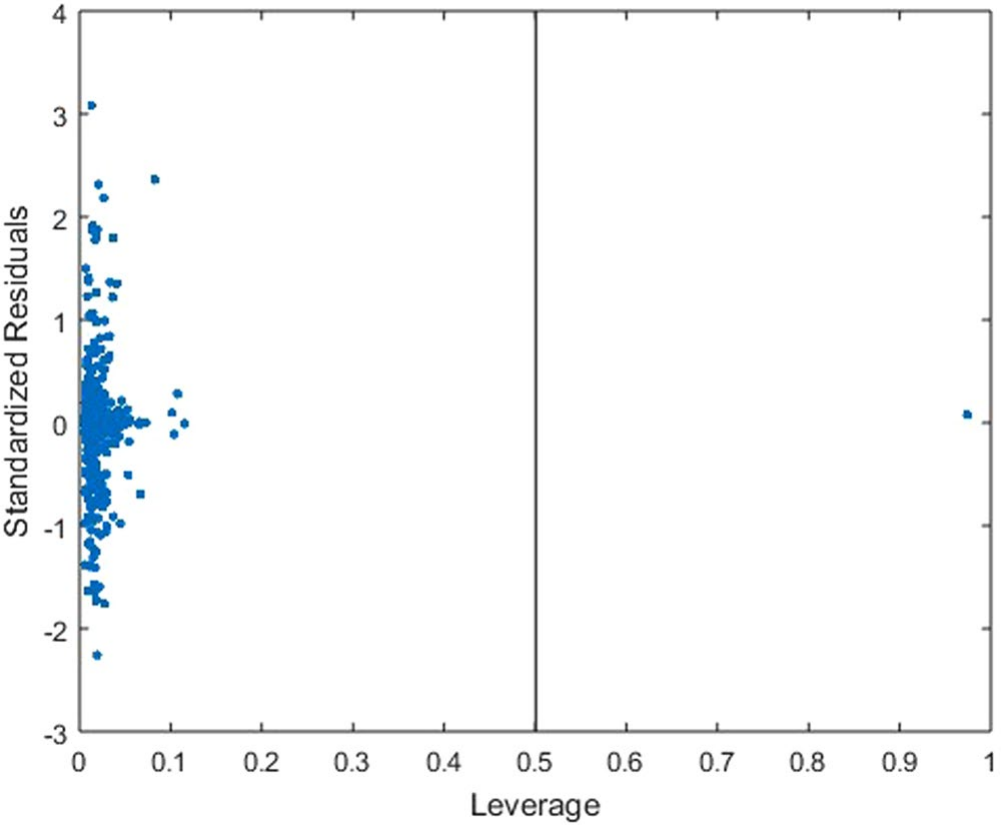
Williams plot for the ALO-ANFIS model with h* = 0.5.

The applicable domain for the proposed ALO-ANFIS QSAR model was determined using the Williams graph shown in Fig. 8, where the leverage value and standardized residuals are plotted. Figure 8 shows that one molecule has a *h* value higher than h^*^ = 0.5, which indicates that this molecule represents an outlier for the structure; therefore, the predicted values for this molecule should be considered as being extrapolated using the ALO-ANFIS QSAR model.

### Comparison with other models

The performance of the proposed QSAR model is compared with other two models namely, PSO-ANFIS and GA-ANFIS (see the supplementary file for more information about PSO and GA) as given in Table 4. From this table it can be concluded that the PSO-ANFIS model is better than GA-ANFIS model in terms of *R*^2^ and *RMSE* for both training and testing sets. However, the proposed QSAR model is still better than the other two models except the *R*^2^ for training set the PSO-ANFIS is better. In addition, the ALO-ANFIS selects smaller number of descriptors than the other models with less times computational.

**Table 4:** The comparison results between the QSAR models.

QSAR model	GA-ANFIS		PSO-ANFIS		ALO-ANFIS	
Statistics value	Training set	Testing set	Training set	Testing set	Training set	Testing set
*R* ^2^	0.948	0.855	0.953	0.898	0.952	0.923
*RMSE*	0.298	0.987	0.256	0.911	0.1104	0.588
Time(s)	398.920		225.129		198.939	
No. Descriptors	19		16		9	

**Figure 9:**
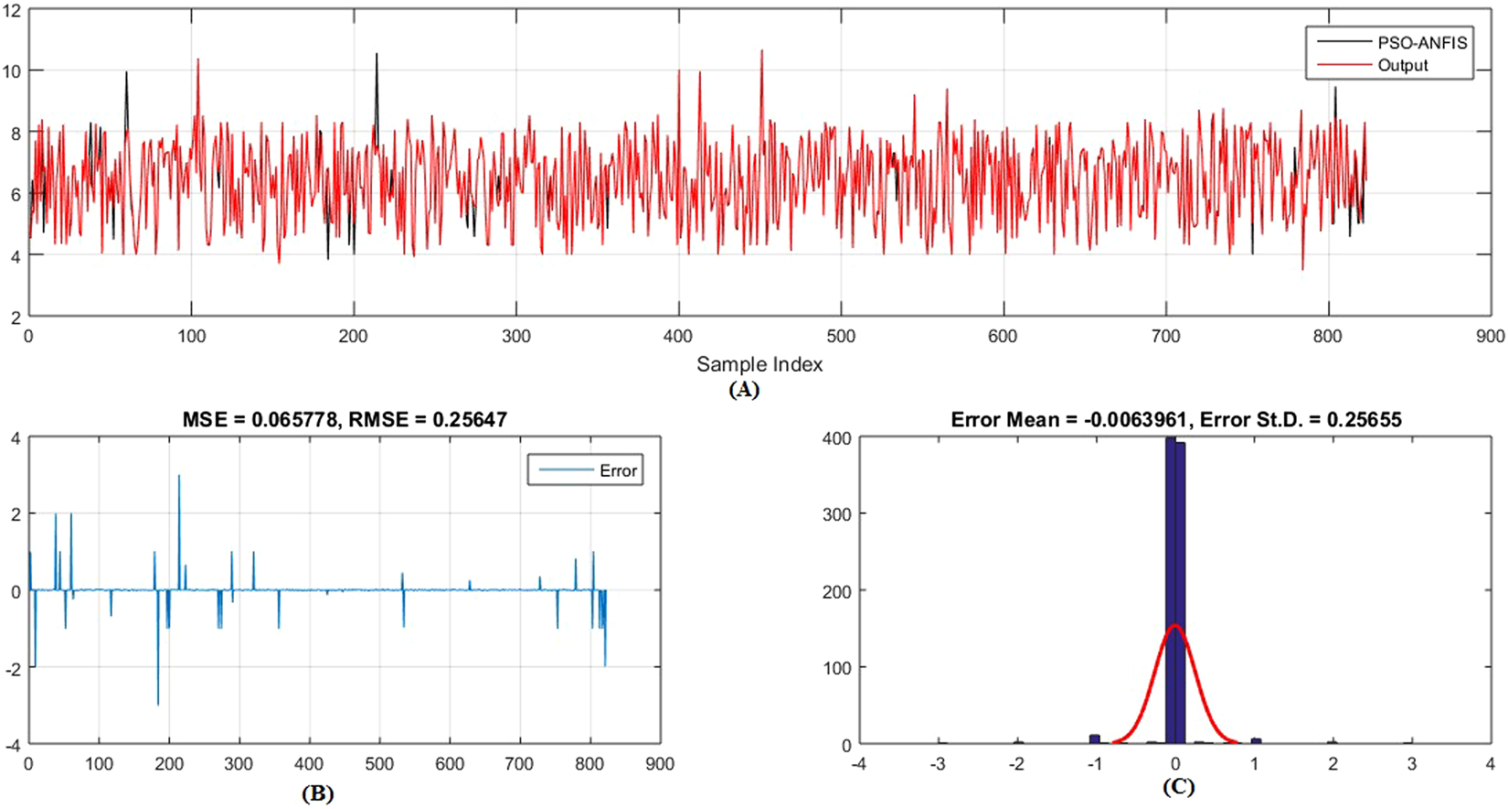
Training set results obtained by the PSO-ANFIS QSAR model (the output in the legend refer to the actual testing set), (**A**) the predicted versus the actual, (**B**) the MSE and RMSE values, (**C**) the histogram of the Error.

**Figure 10:**
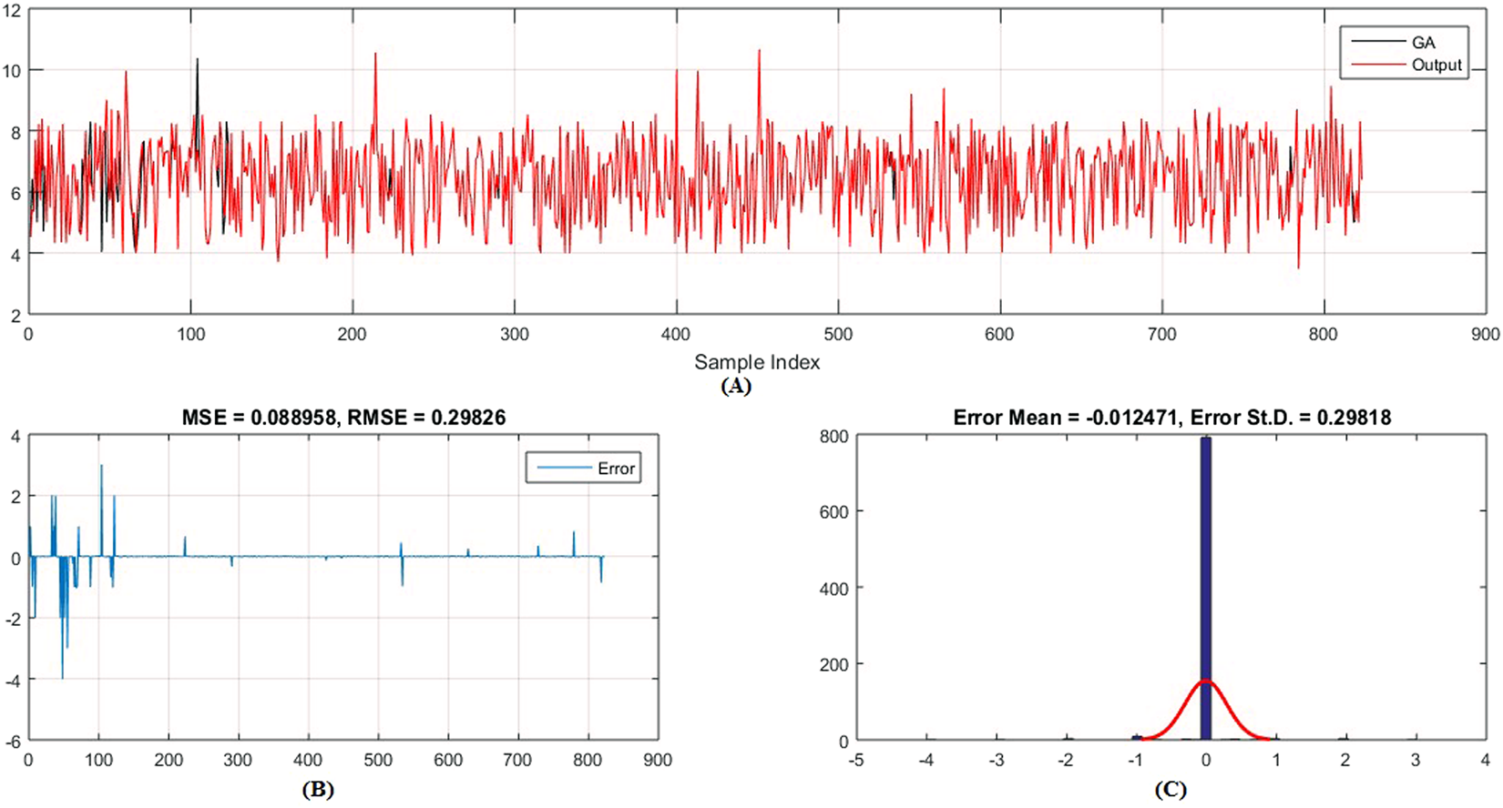
Training set results obtained by the GA-ANFIS QSAR model (the output in the legend refer to the actual testing set), (**A**) the predicted versus the actual, (**B**) the MSE and RMSE values, (**C**) the histogram of the Error.

**Figure 11:**
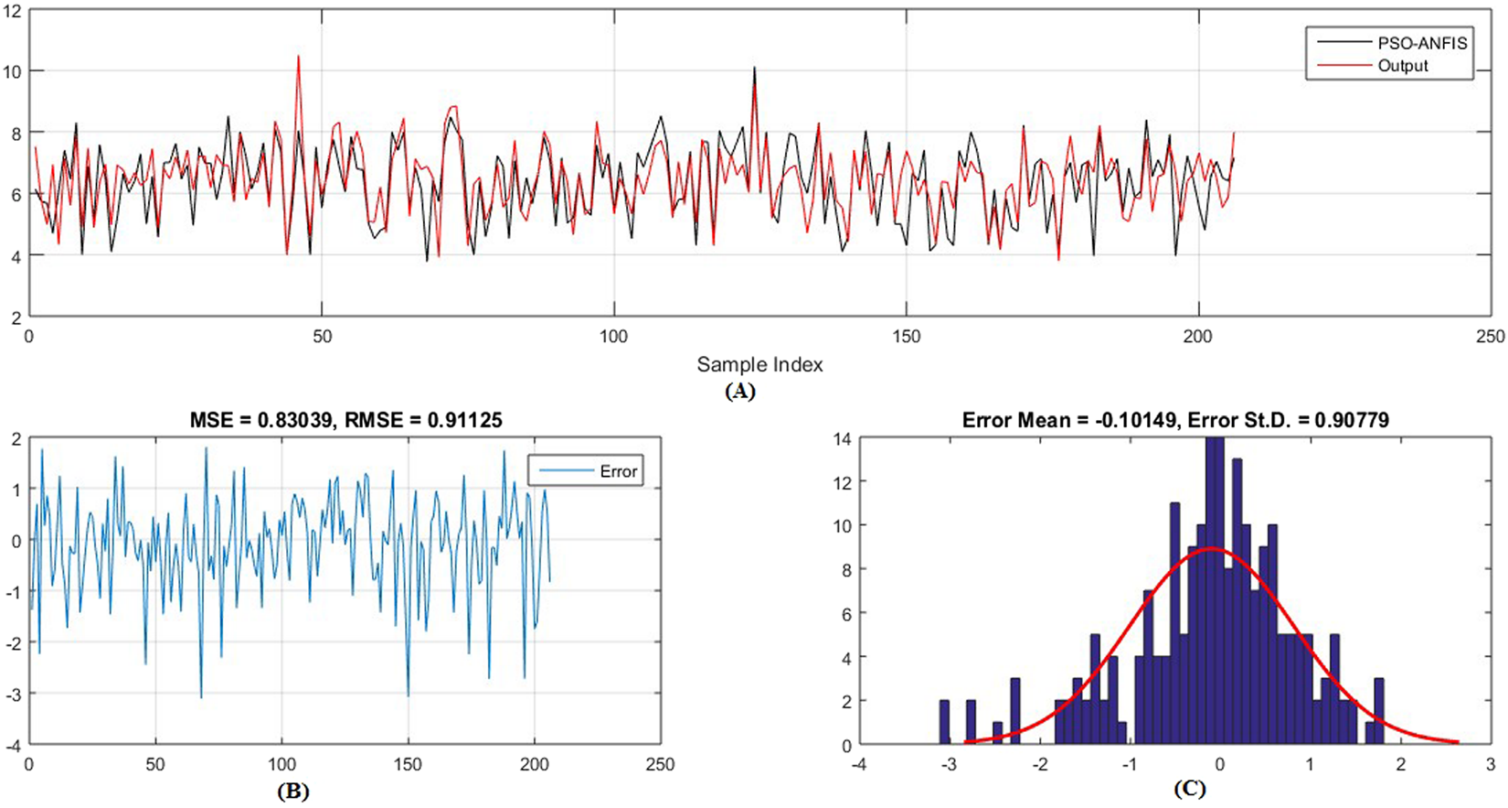
Testing set results obtained by the PSO-ANFIS QSAR model (the output in the legend refer to the actual testing set), (**A**) the predicted versus the actual, (**B**) the MSE and RMSE values, (**C**) The histogram of the Error.

**Figure 12:**
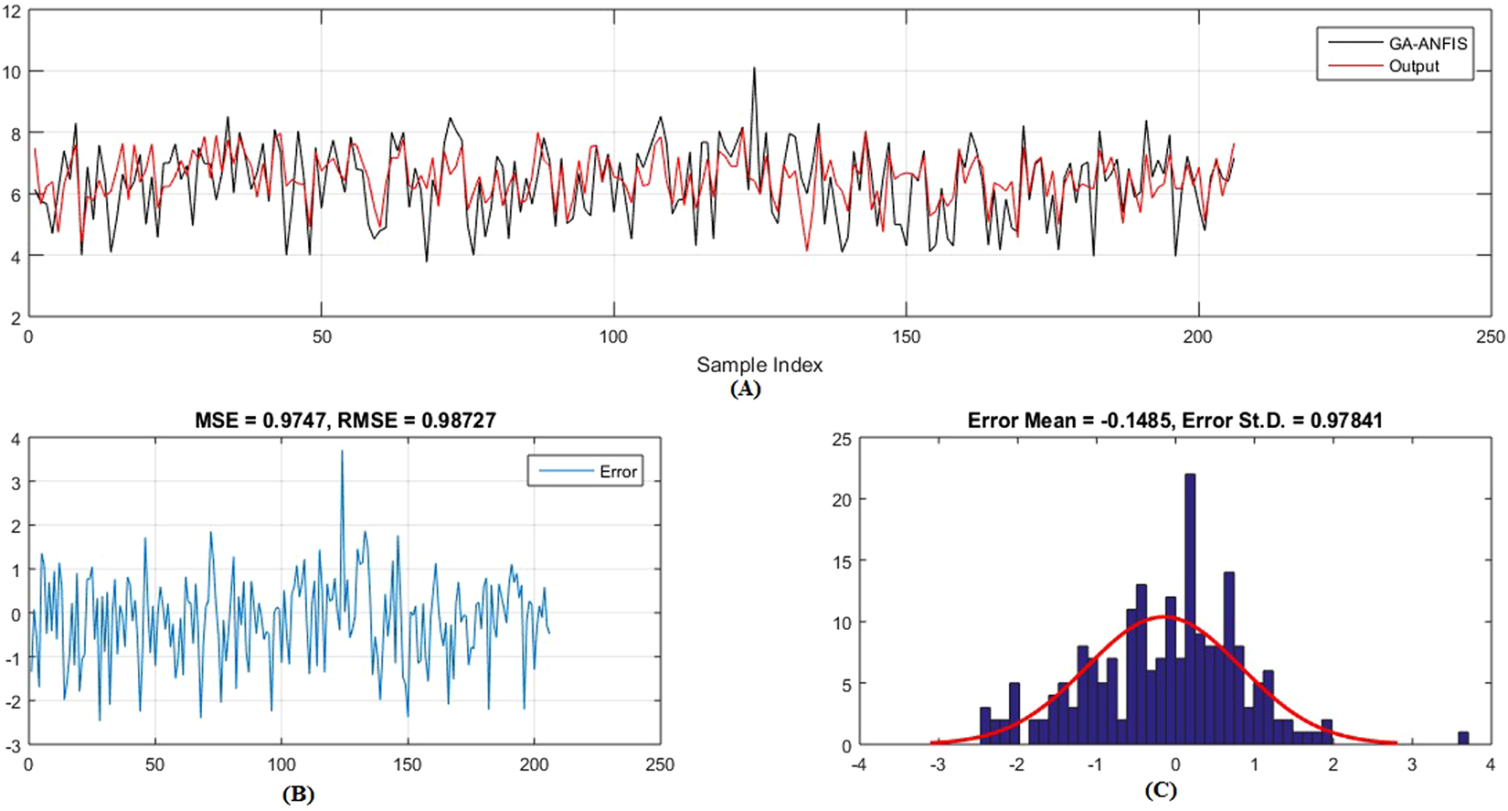
Testing set results obtained by the GA-ANFIS model (the output in the legend refer to the actual testing set), (**A**) the predicted versus the actual, (**B**) the MSE and RMSE values, (**C**) The histogram of the Error.

Moreover, (Figs 9–12) show the actual PIC_50_ value versus the predicted values by using PSO-ANFIS and GA-ANFIS, respectively, for training set (testing set). From these figures we can observed that the PSO-ANFIS is closed to the actual values than the GA-ANFIS, especially, in the testing set this goodness of fit is appeared.

From all the pervious results we can conclude that the proposed ALO-ANFIS model is better than the other two models in terms of performance measures (*R*^2^, *RMSE* Time(s), No. Descriptors).

From the previous results we concluded that the 9 features that selected by the proposed QSAR model have the largest influencing the HCVNS5B inhibitors. However, the proposed model cannot write its output as a regression equation to describe the relation between the 9 descriptors and the PIC_50_, so, it is difficult to interpret the obtained results. However, the harmony between these descriptors create potent HCV inhibitors as the following:

**Molecular flexibility:** The word flexibility is depending on the chemical graph and refers to the molecular mass, branches, rings and heteroatoms. The importance of molecular flexibility in chemistry and biology are many. As in receptor-ligand interactions, the flexible molecules near pharmacophores may led to reaction inhibition and in chemical reactions, it may led to intermolecular interactions and consequently physical changes^62^.

**Molecular complexity:** It represents the sum of bond connectivity’s of molecular structures. It is a simple tool to design a synthetic pathway to a specific molecule. The relation between molecular complexity and biological activity is mentioned before^63^, it is noticed that more simple molecular structure will produce more potent drug^64^.

**Hydrogen bonding**: Is a weak bond which formed between hydrogen atom and an electronegative atom like Oxygen, nitrogen and sulfur. There are two types of Hydrogen bonds, if it is formed between molecules, it will be named intermolecular or within a molecule and it will be called intramolecular^65^.

**Number of rotatable bonds (nrotb):** It represents the change in conformational entropy of a molecule^66^. It also express molecular flexibility and oral bioavailability of drugs^67^. Searle and Williams revealed that each rotatable bond introduces about 1.2–1.6 kcal/mol in changing of binding free energy, assuming complete loss of rotational freedom^68^.

**Rings and Aromatic Rings:** Debnath *et al*.^69^, noticed that increasing hydrophobicity is in linear relation with mutagenic effect.

**Symmetric atoms:** It represents 3D property and it describes atom distribution with respect to some invariant reference frames^70^.

Finally, from the previous discussion it can be observed that the proposed ALO-ANFIS QSAR model provides an efficiency and effectiveness. Since it can selecting an optimal subset of descriptors that increase the regression accuracy. This promising results are achieved due to the ALO algorithm has good ability to balance between the exploration and exploitation during the search process about the optimal solution. Also, due to the good properties of the ANFIS model to solve the QSAR regression problem, since it combines ANN and fuzzy logic system.

### Molecular docking and QSAR

**Figure 13:**
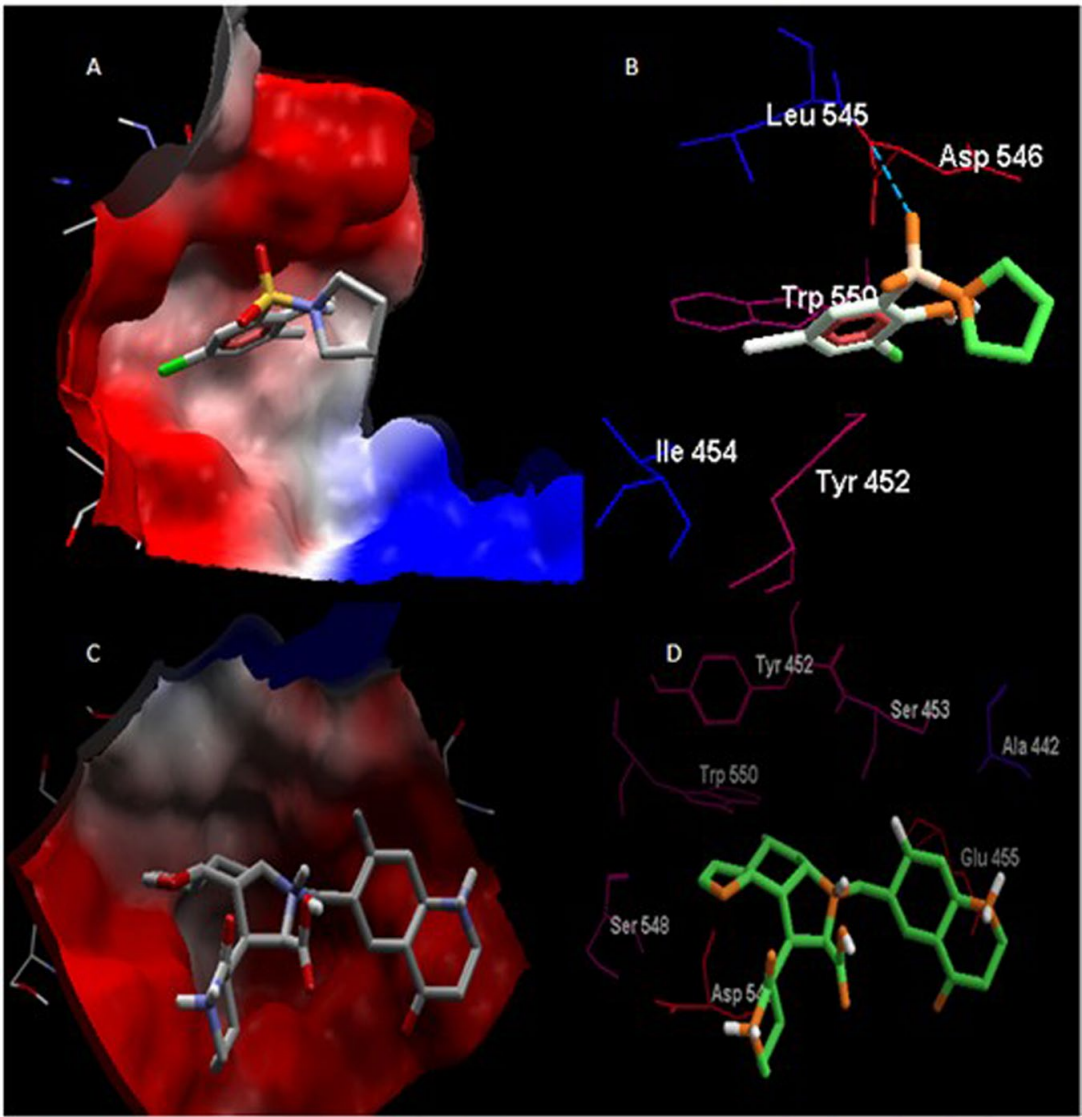
The lowest and best hit in the current docking set were shown in hit (**A**–**D**) respectively.

In Fig. 13, Amino acids were colored according to hydropathy while ligand according to atom type in electrostatic interactions in A and C view. Also ligand was colored according to hydrophobicity in B and D image segments. Hydrogen bond was represented in blue dashed line. The size of compound in C is more accommodate to protein binding site than A, this explain A(the lowest hit) with binding energy = −5.1 kcal/mol and C (the best hit) with binding energy = −13.5 kcal/mol.

**Figure 14:**
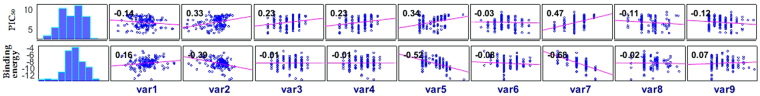
The correlation between the PIC_50_, Bending energy and the 9 descriptors.

In the current study, the highly potent and selective molecular compounds^56^ were depend on several descriptors like molecular flexibility (var1), molecular complexity (var2), non-C/H atoms (var3), electronegative atoms (var4), stereo centers (var5), rotatable bonds (var6), rings (var7), aromatic atoms (var8) symmetric atoms (var9), so a curve will be made between these descriptors versus binding energy and PIC50 as in Fig. 14. From this figure, it can be observed that the value of the PIC_50_ is increased with increase the value of the following descriptors (var2, var3, var4, var5, and var7), however, with the other descriptors its value is decreased, with small value, by increasing their value. In addition, the value of binding energy is decreased with decreasing the value of var2, var5, and var7 descriptors, and increased with other descriptors.

## Conclusion

HCV NS5B is an interesting target for antiviral therapy with limited side effects and it has been the subject of extensive trials to develop nucleoside and non-nucleoside inhibitors. In this study, we used a series of HCV NS5B inhibitors to build a QSAR model. The chemical descriptors were calculated using the DataWarrior package. However, any QSAR model is usually influenced by the number of descriptors and the regression method employed. Therefore, in this study, we developed a new QSAR model for assessing inhibitors and non-inhibitors of HCV NS5B. The proposed model comprised two stages, where we used the ALO algorithm to determine the most relevant descriptors related to the PIC_50_ values in the first stage, and ANFIS was then used to determine the nonlinear relationships between the selected descriptors and the PIC_50_ values in the second stage. The results obtained by the proposed model indicate that it is an acceptable approach for predicting the activity of drugs as HCV NS5B inhibitors.

According to the promising results obtained using the proposed model, we will apply this method to other complex problems in drug design in future research, as well as other applications such as wind speed prediction by making suitable improvements.

## Electronic supplementary material

Supplementary Information

Supplementary Dataset
