# Supplementary material for: Quantitative Structure-Activity Relationship Model for HCVNS5B inhibitors based on an Antlion Optimizer-Adaptive Neuro-Fuzzy Inference System
Source: Sci Rep. 2018 Jan 24;8:1506. doi: 10.1038/s41598-017-19122-y (PMC5784174; doi:10.1038/s41598-017-19122-y)
Supplement: Supplementary file 1 — Supplementary Information [file 41598_2017_19122_MOESM1_ESM.docx]

Quantitative Structure-Activity Relationship Model for HCVNS5B inhibitors based on an Antlion Optimizer-Adaptive Neuro-Fuzzy Inference System

**Mohamed Abd Elaziz^1,*^, Yasmine S. Moemen^2^, Aboul Ella Hassanien^3^, Shengwu Xiong ^4,*^**

1. **Particle swarm optimization**

Kennedy and Eberhart [1] was proposed the Particle swarm optimization (PSO), in which it simulates the social behavior of group animals to share the private information about the source of foods. The position of each member of this group represent the solution of the given problem.

The PSO algorithm starts by constructing a random position for the particles ($x_{i}$) and assign a random velocity$\left( v_{i} \right)$to each particle $x_{i}$ in a dimension $j$-th.

Then the fitness function is computed for each particle$x_{i}$. After that, the velocity of each particle $v_{i}$ is updated using the following equation

$$v_{ij}^{t+1}=wv_{ij}^{t}+c_{1}r_{1}{(x}_{ij}^{p(t)}-x_{ij}^{t})+c_{2}r_{2}{(x}_{j}^{g\left( t \right)}-x_{ij}^{t}) (S1)$$

Where $w,c_{1}{,r}_{1},c_{2},$and $r_{2}$ are random numbers belong to the interval $[0,1]$, $w$ represents an inertia weight and used to enhance the convergence speed; $c_{1}$and $c_{2}$ are a constants that represents the acceleration coefficients.

The position $x_{i}$will be updated using its velocity $v_{i}$ as

$$x_{i}=x_{i}+v_{i} (S2)$$

The fitness function of each $x_{i}$ is cimputed and the best global solution is determined also, the best personal position. The previous two steps (update the velocity and position; and compute the fitness function) are repeated until the convergence condition are reached.

1. **Genetic algorithm**

Genetic algorithm (GA)[2] is evolutionary method that simulates the process of selecting and developed the genetics. The GA begins by generating a random population of chromosomes that represent the solutions for the given problem. These solutions are updated using three operators called selection, crossover, and mutation which aim to generate a new offsprings that carry the good characteristics of their parents. This operation (create offsprings) are repeated until predefined stop condition is met.

The performance of solutions are evaluated using the fitness function, and better solutions are selected for generating a new solutions through the crossover and mutation processes.

The crossover operator is used to exchange the genes between two solutions $S_{i}$ and $S_{j}$ according to propability as:

| $S_{i}=[s_{i1},s_{i2},\ldots,s_{ik},\ldots, s_{id}]$ | Using propability $p\in[0,1]$ | $S_{i}^{'}=[s_{i1},s_{i2},\ldots,s_{jk},\ldots, s_{jd}]$ |
| --- | --- | --- |
| $S_{j}=[s_{j1},s_{j2},\ldots,s_{jk},\ldots, s_{jd}]$ |  | $S_{j}^{'}=[s_{j1},s_{j2},\ldots,s_{ik},\ldots, s_{id}]$ |

Where $S_{i}^{'}$ and $S_{j}^{'}$ are the solutions after crossover. $k$ represents a random integer between 1 and the dimension $d$.

The mutation operator is defined as:

$$S_{i}^{'}=\left[ s_{i1}^{'},s_{i2}^{'},\ldots, s_{id}^{'} \right]===> S_{i}^{'}=\left[ s_{i1}^{'},s_{id}^{'},\ldots, s_{i2}^{'} \right] (S3)$$

## Where the Exchange Mutation is used, in which a two genes are selected randomly and exchange

Reference

1. Kennedy, J.; Eberhart, R. ["Particle Swarm Optimization"](http://ieeexplore.ieee.org/stamp/stamp.jsp?arnumber=488968). Proceedings of IEEE International Conference on Neural Networks.**IV,** 1942–1948(1995).
2. Arezoo Sarkheyli, Azlan Mohd Zain, Safian Sharif. Robust optimization of ANFIS based on a new modified GA. Neurocomputing. **166,** 357–366 (2015).
